# Supplementary material for: Generation and Identification of the Number of Copies of Exogenous Genes and the T-DNA Insertion Site in SCN-Resistance Transformation Event ZHs1-2
Source: Int J Mol Sci. 2022 Jun 20;23(12):6849. doi: 10.3390/ijms23126849 (PMC9245598; doi:10.3390/ijms23126849)
Supplement: Supplementary file 1 [file ijms-23-06849-s001.zip › Supplement Figure S1 and S2-ijms 1749415.pdf]

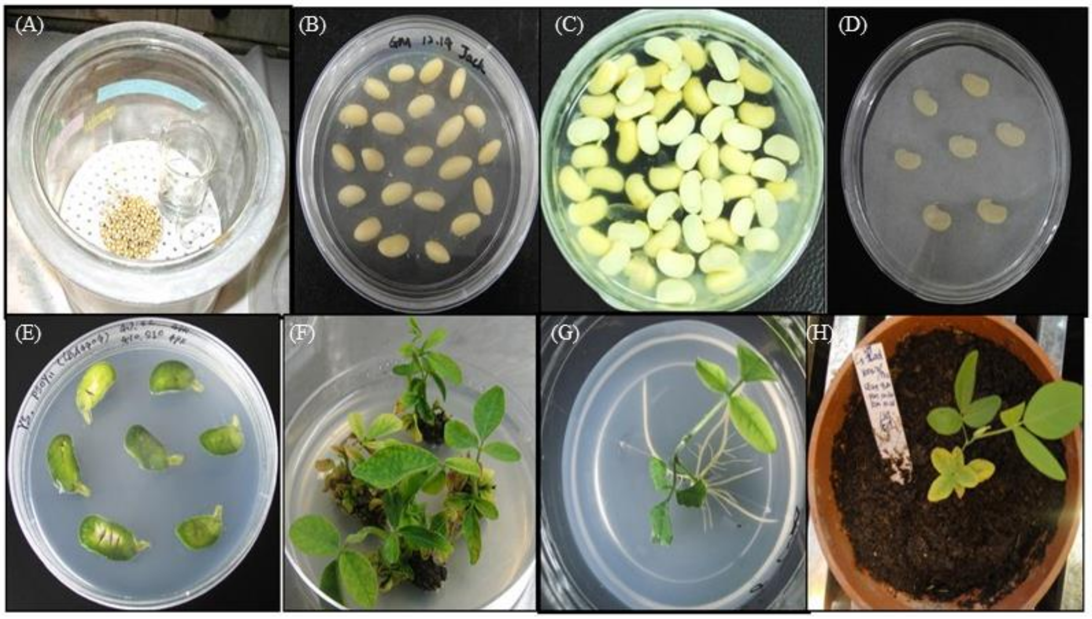


Figure S1 Schematic diagram of the *Agrobacterium-tumefaciens* mediated transformation method in soybean. (A): Seed sterilization by chlorine gas; (B) Seed germination on the germination medium; (C) Isolating 1-2 d germinated cotyledonary explants; (D) Explants inoculating with *Agrobacterium*; (E) Explants co-cultivating with *Agrobacterium*; (F): Explants on the shoot induction medium; (F): Shoot elongation on the shoot elongation medium; (G): Shoot rooting; (H): Plantlet domestication and cultivation.

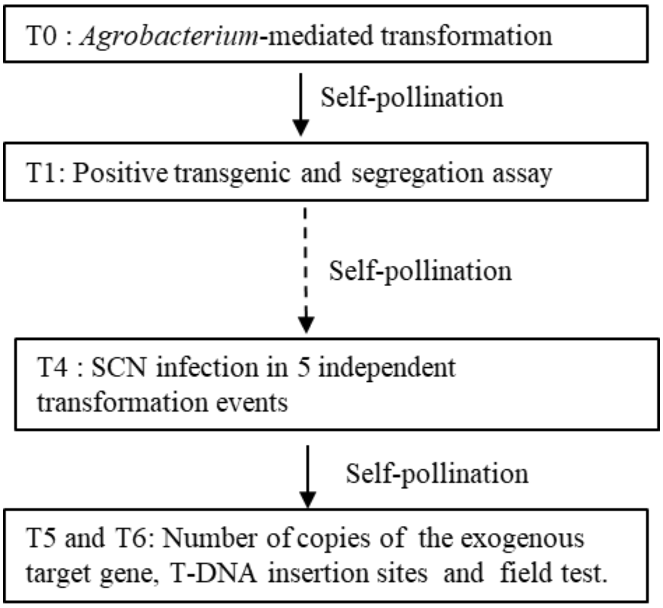


Figure S2 Generation of transgenic progeny and assay index in different progeny.T0, T1, T4, T5 represented different transgenic progeny.
